# Genotypic and phenotypic characterization of *Staphylococcus aureus* isolated from human milk of asymptomatic women or women with acute mastitis

**DOI:** 10.1128/msystems.00797-25

**Published:** 2025-08-15

**Authors:** Rubén Jurado, Alberto Aragón, Natalia Hernando, Josué Jara, Juan Miguel Rodriguez, Belén Orgaz, Leonides Fernández

**Affiliations:** 1 Sección Departamental de Farmacia Galénica y Tecnología Alimentaria, Facultad de Veterinaria, Universidad Complutense de Madrid 16734, Madrid, Spain; 2 Instituto Pluridisciplinar, Universidad Complutense de Madrid 16734, Madrid, Spain; 3 Sección Departamental de Nutrición y Ciencia de los Alimentos, Facultad de Veterinaria, Universidad Complutense de Madrid 16734, Madrid, Spain; NYU Langone Health, New York, New York, USA

**Keywords:** *Staphylococcus aureus*, mastitis, human milk, genome mining, virulence, antibiotic resistance

## Abstract

Acute mastitis is a common infection during lactation, primarily caused by *Staphylococcus aureus*, a bacterium known for its ability to form biofilms within mammary ducts and develop antibiotic resistance. This study aimed to genomically characterize *S. aureus* strains isolated from women with acute mastitis and healthy asymptomatic women to better understand how *S. aureus* strains transition from harmless components of the human milk microbiota to pathogenic agents responsible for mastitis. Whole-genome sequencing was performed on nine *S*. *aureus* strains—six from women with mastitis and three from healthy women—followed by *in silico* analyses of core and accessory genes, resistome, virulome, mobilome, and secondary metabolite synthesis to identify genes related to virulence, antibiotic resistance, biofilm formation, and mobile genetic elements such as plasmids, bacteriophages, and pathogenicity islands. Antimicrobial resistance profiles were evaluated using Sensititre EUST2 plates, antimicrobial activity by an agar diffusion method, biofilm formation in 96-well plates, and siderophore production with the Chrome Azurol S assay. Results showed that complete bacteriophage genomes were only present in *S. aureus* strains isolated from mastitis cases. Some virulence genes, including *fnbB* and *cna*, were absent in strains from healthy women. Both types of *S. aureus* strains exhibited biofilm formation capacity, with mastitis-associated strains SA4 and SA5 being the highest biofilm producers. Similarly, although all strains secreted siderophores, SA4 and SA55 exhibited the strongest siderophore production, indicating a link between this trait and virulence in mastitis-associated strains. The analysis of key genomic features, including virulence factors, resistance genes, and biofilm-forming capabilities, revealed some mechanisms by which *S. aureus* contributes to the pathogenesis of mastitis.

**IMPORTANCE:**

Acute mastitis is a widespread infection in lactating women, and its main cause, *Staphylococcus aureus*, has developed resistance to antibiotics, making treatment challenging. The ability of this bacterium to form biofilms complicates its eradication from the mammary glands. Understanding the genomic and phenotypic characteristics of *S. aureus* strains associated with mastitis, compared to those isolated from asymtomatic women, is critical for developing better treatment strategies. This study provides new insights into the genetic features, such as virulence factors, antibiotic resistance profiles, and presence of bacteriophages, that make *S. aureus* strains pathogenic in mastitis. It also highlights the potential of biofilm formation and siderophore production as key factors in mastitis progression. These findings could guide the development of novel therapeutic approaches, such as targeted therapies or probiotics, which can more effectively treat mastitis and reduce reliance on antibiotics, ultimately improving maternal and infant health.

## INTRODUCTION

*Staphylococcus aureus* is a relatively common microorganism in human mucosal surfaces, predominantly in those of the gut and upper respiratory tract, with a prevalence of approximately 25% and 30%, respectively, in asymptomatic individuals (1, 2). However, asymptomatic colonization by *S. aureus* has long been recognized as a significant risk factor for invasive infections (3, 4); in fact, *S. aureus* is one of the world’s leading causes of infection-related mortality being the only bacterial pathogen, together with *Mycobacterium tuberculosis*, consistently exceeding rates of one million deaths annually (5).

Infections caused by *S. aureus* vary from uncomplicated mild skin and soft tissue infections to life-threatening conditions (5–7). The ability of *S. aureus* to cause such a diverse array of infections has been attributed to its extensive arsenal of protein and non-protein elements that facilitate host colonization, virulence determinants, and immune evasion mechanisms (8–10). The clinical impact of these infections is often exacerbated by the presence of antimicrobial resistance, further complicating treatment and management (11–13). Among *S. aureus* infections, acute mastitis is the most prevalent among lactating women.

Human milk may contain *S. aureus,* although its frequency and concentration vary depending on the breast health of the host, from low or very low in asymptomatic healthy women to high or very high in women with acute mastitis (14–16). It means that, although human milk harbors different compounds with the ability to protect the breast-fed infant against staphylococcal infections (17–20), human milk may be a source of *S. aureus* to the infant gut (21, 22). At present, the precise mechanisms underlying the transition from commensalism to pathogenicity remain poorly understood (23); therefore, the objective of this study was to genomically characterize *S. aureus* strains isolated from milk samples collected from both mastitis-affected and healthy asymptomatic women to gain deeper insights into how *S. aureus* strains may evolve from non-pathogenic members of the human milk microbiota to mastitis-causing pathogens. To achieve this purpose, the genomes of nine *S*. *aureus* strains (six isolated from milk of women with acute mastitis and three from that of healthy asymptomatic women) were sequenced, and comprehensive *in silico* analyses were performed to investigate their core and accessory genome, resistome, virulome, mobilome, and secondary metabolite synthesis capacity. Some of the key findings relevant to human mastitis were further validated through phenotypic assays.

## MATERIALS AND METHODS

### Bacterial strains and growth conditions

Nine *S*. *aureus* strains were used in this study. All of them were isolated previously and identified by 16S ribosomal RNA (rRNA) gene sequencing (14). Six of the strains had been isolated from the milk of women suffering from mastitis (SA1, SA3, SA4, SA5, SA35, and SA55, originally coded as S3B, OD111, DH3a, O2LI2, ACLI1, and BALD1, respectively) and three from the milk of clinically healthy women (SA7, SA14, and SA15, originally coded as L1741, LC001, and LX153b, respectively). Unless otherwise specified, *S. aureus* strains were cultured in brain heart infusion (BHI) medium (Oxoid, Basingstoke, UK). All cultures were incubated aerobically at 37°C for 24 h under static conditions, from stock cultures maintained in glycerol (1.5%, vol/vol) at −80°C.

### Whole genome sequencing, assembly, and annotation

For genomic DNA extraction, 1 mL of overnight *S. aureus* cultures was centrifuged at 5,000 × *g* for 10 min and the pellets were suspended in 180 µL of lysis buffer containing 20 mM Tris-HCl (pH 8.0), 2 mM EDTA, 1.2% Triton X-100, lysozyme (20 mg/mL), and mutanolysin (10 U/µL). Cell suspensions were enzymatically treated with 4 µL of RNase A (100 mg/mL) for 5 min and then with 25 µL of proteinase K (20 mg/mL) at 56°C for 30 min. After this treatment, genomic DNA was extracted with the QIAamp DNA Mini Kit (QIAGEN, Hilden, Germany), according to the manufacturer’s instructions for Gram-positive bacteria. Genomic DNA sequencing was performed using Illumina HiSeq technology with a 250 bp paired-end protocol and 30× coverage by MicrobesNG (Birmingham, UK). FastQC (version 0.11.9) (24) was used to evaluate the quality of the sequence reads, and FastP (version 0.23.2) (25) was used to exclude low-quality reads and adapters. High-quality reads were assembled using SPAdes (version 3.13.1) (26) with k-mer sizes of 55, 75, and 97. Genome assembly quality was further evaluated using QUAST (version 5.0.2) (27), a tool that assesses the quality of genome assemblies by calculating metrics such as N50 and L50, which represent contig length distribution and assembly continuity, respectively. Bacterial species identification was confirmed *in silico* using Kraken2 (28). Assembled contigs were annotated with RAST (29, 30) and Prokka (version 1.14.6) (31), which provided the number of RNA genes and coding sequences (CDSs). The draft genome sequences of the nine *S*. *aureus* strains sequenced in this study were deposited in NCBI under BioProject ID PRJNA1200689.

### Genotyping and comparative genomics

To reconstruct the evolutionary relationships among the strains, a core genome single-nucleotide polymorphism (SNP) phylogenetic analysis was performed. High-quality SNPs were identified using Snippy (version 4.6.0) (32), which maps contigs from each isolate against the reference genome *Staphylococcus aureus* NCTC8325 and calls variants. The resulting SNP alignments were processed with Snippy-core to extract a core genome alignment shared among all isolates. This alignment was used as input for RAxML (version 8.2.12) (33) to construct a maximum likelihood phylogenetic tree under the GTR+GAMMA model with 1,000 bootstrap replicates. The final tree was visualized using Interactive Tree Of Life (34).

Multilocus sequence typing (MLST) was performed using MLST 2.0 (35) (https://cge.food.dtu.dk/services/MLST/). OrthANI (36) (https://www.ezbiocloud.net/) was used to compare the average nucleotide identity (ANI) of genomic strains. Visualization of multiple genome comparisons was conducted using BLAST Ring Image Generator (BRIG) (37). Roary (version 3.13.0) (38) was used to identify core and accessory genes, to build a presence/absence matrix, and to cluster genes to allow gene content comparison across multiple genomes. This analysis was improved with Spine and AGEnt (39) to define and characterize the core and the accessory genome, respectively. With these tools, the complete nucleotide sequences of both the core and accessory genomes were obtained. To elucidate the functional roles of these genes, BlastKOALA (version 2.2) (40) was used to assign proteins to Kyoto Encyclopedia of Genes and Genomes (KEGG) Orthology groups and the RAST server (29, 30) to annotate genes based on subsystems and metabolic pathways.

Antibiotic resistance genes were identified using Resfinder (version 4.6.0) (41) (http://genepi.food.dtu.dk/resfinder) with a stringent identity threshold of 90% and a coverage threshold of 60%, and the Comprehensive Antibiotic Resistance Database (42) (CARD, https://card.mcmaster.ca/analyze/rgi). The virulence factors were analyzed using two approaches: the virulence factor database (43) (VFDB, http://www.mgc.ac.cn/VFs/), using *S. aureus* subsp. *aureus* NCTC 8325 (NCBI Reference Sequence: NC_007795.1) as the reference genome, and VirulenceFinder 2.0 (44) (https://cge.food.dtu.dk/services/VirulenceFinder/), applying a minimum length threshold of 60% and a sequence identity threshold of 90% for reliable identification. Secondary metabolism-related genes were predicted with BAGEL4 (45) (http://bagel4.molgenrug.nl/) and antiSMASH 7.0 (46) (https://antismash.secondarymetabolites.org/).

The study of mobile genetic elements included the detection of plasmids, phage regions, and pathogenicity islands. First, MOB-suite (version 3.1.9) (47) was used to identify plasmids, which assigns plasmid names based on the “Mash nearest neighbor” approach by comparing sequences against a curated plasmid database. The detected plasmid sequences were then matched against the assembled genomes using BLASTn (48) to confirm their presence. Using PHASTEST (version 3.0) (49) (https://phastest.ca/), phage regions were detected and classified as intact, questionable, or incomplete. To detect pathogenicity islands, *S. aureus* pathogenicity islands already included in the Pathogenicity Island Database (50) (PAI DB, http://www.paidb.re.kr/about_paidb.php) were downloaded and compared with the assembled genomes using BLASTn. Using VFDB (43), VirulenceFinder (44), and CARD (42), the existence of virulence factors and antibiotic resistance genes linked to each mobile genetic element was then examined.

### Antibiotic susceptibility testing

*In vitro* antibiotic susceptibility tests were performed using the microdilution method with Sensititre EUST2 plates (Thermo Fisher Scientific, Cleveland, OH, USA). For this, 3–5 colonies of each *S. aureus* strain were transferred to 5 mL of saline solution (0.85% NaCl, wt/vol) until achieving a 0.5 McFarland standard (equivalent to ~10^8^ colony-forming units [CFU]/mL). Ten microliters of each standardized bacterial suspension was added to 10 mL of Müeller-Hinton broth (Oxoid, Basingstoke, UK) and, subsequently, 50 µL of these were inoculated into each well of the Sensititre plates (one plate per strain). The antibiotics analyzed were cefoxitin (0.5–16 µg/mL), ciprofloxacin (0.25–8 µg/mL), clindamycin (0.12–4 µg/mL), erythromycin (0.25–8 µg/mL), fusidate (0.25–4 µg/mL), gentamicin (0.5–16 µg/mL), linezolid (1–8 µg/mL), penicillin (0.06–1 µg/mL), quinupristin/dalfopristin (0.5–4 µg/mL), rifampin (0.015–0.5 µg/mL), sulfamethoxazole (64–512 µg/mL), tetracycline (0.5–16 µg/mL), trimethoprim (1–16 µg/mL), and vancomycin (1–8 µg/mL). After 18–24 h of incubation at 37°C, each plate was examined for bacterial growth. The results were interpreted according to the breakpoints defined by the European Committee on Antimicrobial Susceptibility Testing (51) and the Clinical and Laboratory Standards Institute (52).

### Antimicrobial activity of *S. aureus* strains against human milk bacteria

The inhibitory capacity of *S. aureus* strains against 10 different indicator strains, all isolated from milk, was examined by an agar diffusion method. First, an isolated colony of each *S. aureus* strain was inoculated into tubes containing 10 mL of BHI broth and incubated overnight at 37°C. Then, 1 mL of the cultures was centrifuged at 5,000 × *g* for 10 min, and the cell-free supernatants (CFS) were recovered and filtered through 0.22 µm filters. Meanwhile, an isolated colony from each indicator strain was inoculated into 10 mL of its respective growth medium: *Limosilactobacillus reuteri* 13LM2-01, *Ligilactobacillus salivarius* 2LM3-30, *Lactococcus lactis* MG1614, *Limosilactobacillus fermentum* I7, and *Enterococcus faecalis* TAB28 were cultured in MRS broth; while *Staphylococcus aureus* 3LM3-30, *Staphylococcus capitis* 1LM3-02, *Staphylococcus devriesei* 40LM2-41, *Staphylococcus epidermidis* 2LM2-42, and *S. epidermidis* 20LM3-01 were cultured in BHI broth. The next day, 15 mL tubes of soft BHI or MRS agar (0.75% [wt/vol] agar) at 55°C were inoculated with 100 µL of a bacterial suspension (~10⁵ CFU/mL) of each indicator strain and poured into empty Petri dishes. Once the agar layer solidified, wells were made using the wide end of a yellow pipette tip, and 50 µL of the CFS from the *S. aureus* strains were added into the wells. The plates were refrigerated for 2 h and then incubated overnight at 37°C. Finally, the inhibition halos were measured. Experiments were carried out in triplicate.

### Assessment of the adhesion capability and biofilm-forming ability of *S. aureus* strains

A colony of each *S. aureus* strain was incubated in BHI broth at 37°C for 24 h. After this, the culture was centrifuged at 5,000 × *g* for 15 min at 4°C, rinsed with fresh broth, and suspended to reach an optical density at 600 nm of 0.1 (~10^7^ CFU/mL). The suspensions were then diluted 1:100 in BHI broth, and 200 µL of each dilution was added into 10 wells of polypropylene 96-well plates. The plates were incubated at 37°C for 1 h. After this initial incubation, the wells were washed twice with saline solution, and fresh BHI broth was added to the wells. To determine the adhered cell population at t_1_, the contents of three wells were vigorously mixed, serially diluted, and plated for cell counting in BHI agar. Subsequently, the plates were incubated for another 24 h to allow biofilm formation from the cells that had adhered during the first hour. After this second incubation, the wells were washed again with saline solution, and 200 µL of fresh BHI was added to three wells. The contents of these wells were mixed for A_600_ reads, followed by serial dilutions and plating in BHI agar to determine the adhered cell population at t_24_. Bacterial counts were expressed as log_10_ CFU/cm^2^. For the remaining four wells, the bacteria adhered to the surface of the plates were fixed by drying at 37°C for 1 h. After fixation, the biofilms were stained by adding 150 µL of a 2% (wt/vol) crystal violet solution, 1% (wt/vol) ammonium oxalate, and 20% (vol/vol) ethanol (Gram-Hucker’s crystal violet oxalate solution, PanReac AppliChem, Barcelona, Spain). The plates were incubated at room temperature for 15 min. The dye was removed, and the wells were washed three times with distilled water. The plates were left to dry at room temperature, after which 150 µL of 96% (vol/vol) ethanol was added to each well. After 30 min of incubation at room temperature, the A_570_ of the resulting solution was measured to quantify the attached biomass.

### Siderophore production

Overnight *S. aureus* cultures in BHI broth were centrifuged at 5,000 × *g* for 15 min at 4°C, the cell pellet was suspended in saline solution, and the optical density (OD) at 600 nm was adjusted to 0.1 (~10⁷ CFU/mL). These suspensions were diluted 1:100 in PMS_7_Ca liquid medium (53) supplemented with 1% casamino acids (Difco Laboratories, Detroit, MI, USA) as an iron-limited medium to induce siderophore production, and 1 mL of each dilution was inoculated into six wells of a polypropylene 24-well plate. The plate was incubated at 37°C with shaking (180 rpm) for 5 days. At each time point (1, 2, and 5 days), the following procedure was carried out: two wells were used for A_600_ measurements and to determine planktonic cell counts, which were expressed as log_10_ CFU/mL; another two wells were stained with crystal violet for A_570_ measurements, and the remaining two wells were used to quantify the biofilm-forming adhered cell population (expressed as log_10_ CFU/cm^2^). The CFS from these six wells was obtained by centrifugation at 5,000 × *g* for 15 min at 4°C and stored at −20°C for subsequent siderophore quantification.

Siderophore quantification was performed using the classic method employing the Chrome Azurol S (CAS) assay described by Schwyn and Neilands (54). The assay was adapted for 96-well plates, where 100 µL of CFS or appropriate controls were mixed with 100 µL of CAS reagent, with two technical replicates for each biological replicate. After incubation at room temperature for 30 min, A_630_ was measured. The percentage of siderophore production was calculated using the following formula:


$$Siderophore\;production\;(\%)\;=\;\left(\frac{A_{\text{blank}}-A_{\text{sample}}}{A_{\text{blank}}}\right)\times 100$$

### Statistical analysis and data visualization

Statistical analysis was carried out using Statgraphics Centurion 19 software (Statistical Graphics Corporation, Rockville, MD, USA). A one-way analysis of variance (ANOVA) was conducted to assess differences in biofilm formation capacity across the different *S. aureus* strains. When significant differences were found, a *post hoc* Tukey-HSD test was applied to compare the means of the groups, with significance determined at the 95% confidence level (*P* < 0.05). Data visualization, including heatmaps and bar graphics, was generated using pheatmap (55) and ggplot2 (56) packages in RStudio version 4.3.1. In the case of heatmaps, hierarchical clustering was performed using Euclidean distance and complete linkage, which are the default settings of the pheatmap function. For correlation analysis, the corrplot (57) package was used, which calculates Pearson’s correlation by default. 

## RESULTS

### Genome assembly, annotation, and phylogenetic analysis of *S. aureus* strains

In this study, nine *S*. *aureus* strains isolated from milk samples, including six from women suffering lactational mastitis (SA1, SA3, SA4, SA5, SA35, and SA55) and three from clinically healthy women (SA7, SA14, and SA15), were sequenced. The genome and assembly characteristics of the sequences, including genome size, guanine-cytosine content, number of contigs, largest contig, N50, and L50, are shown in Table 1. The genome sizes of the isolates ranged from 2.71 to 2.85 Mbp, with a GC content between 32.65% and 32.77%. The number of contigs varied between 29 and 71, with the largest contig covering 285,517 to 999,011 bp. The N50 and L50 values, which represent contig length distribution and assembly continuity, ranged from 102,271 to 696,172 bp and from 2 to 9, respectively. Between 2,455 and 2,678 CDSs were found in each isolate. Regarding RNA genes, in each isolate, 6 to 8 were rRNAs, 50 to 53 were transfer RNAs (tRNA), and one was tmRNA (Table 1).

**TABLE 1:** Genome and assembly characteristics of nine sequenced *S. aureus* strains in the study

Isolate	Genome length (bp)	GC content (%)	No. of contigs (>200 bp)	Largest contig (bp)	N50^*a*^ (bp)	L50^*b*^ (bp)	No. of CDSs	No. of rRNAs	No. of tRNAs	No. of tmRNAs
SA1	2,771,782	32.71	29	698,006	696,172	2	2,564	8	61	1
SA3	2,769,689	32.66	63	336,701	171,622	6	2,543	6	53	1
SA4	2,851,111	32.68	71	285,517	102,271	9	2,678	8	60	1
SA5	2,789,636	32.74	47	343,878	120,275	9	2,590	7	60	1
SA7	2,813,524	32.77	35	676,725	349,595	6	2,633	7	59	1
SA14	2,813,524	32.77	37	999,011	421,138	4	2,471	8	60	1
SA15	2,710,679	32.70	57	383,449	170,877	4	2,455	8	61	1
SA35	2,770,208	32.65	30	437,631	244,027	3	2,576	7	58	1
SA55	2,730,657	32.66	31	617,203	244,030	2	2,526	8	61	1

^*a*^ N50: length of the shortest contig in the set of longest contigs that together cover at least 50% of the assembled genome.

^*b*^ L50: number of contigs required to cover at least 50% of the total length of the assembled genome.

**Fig 1:**
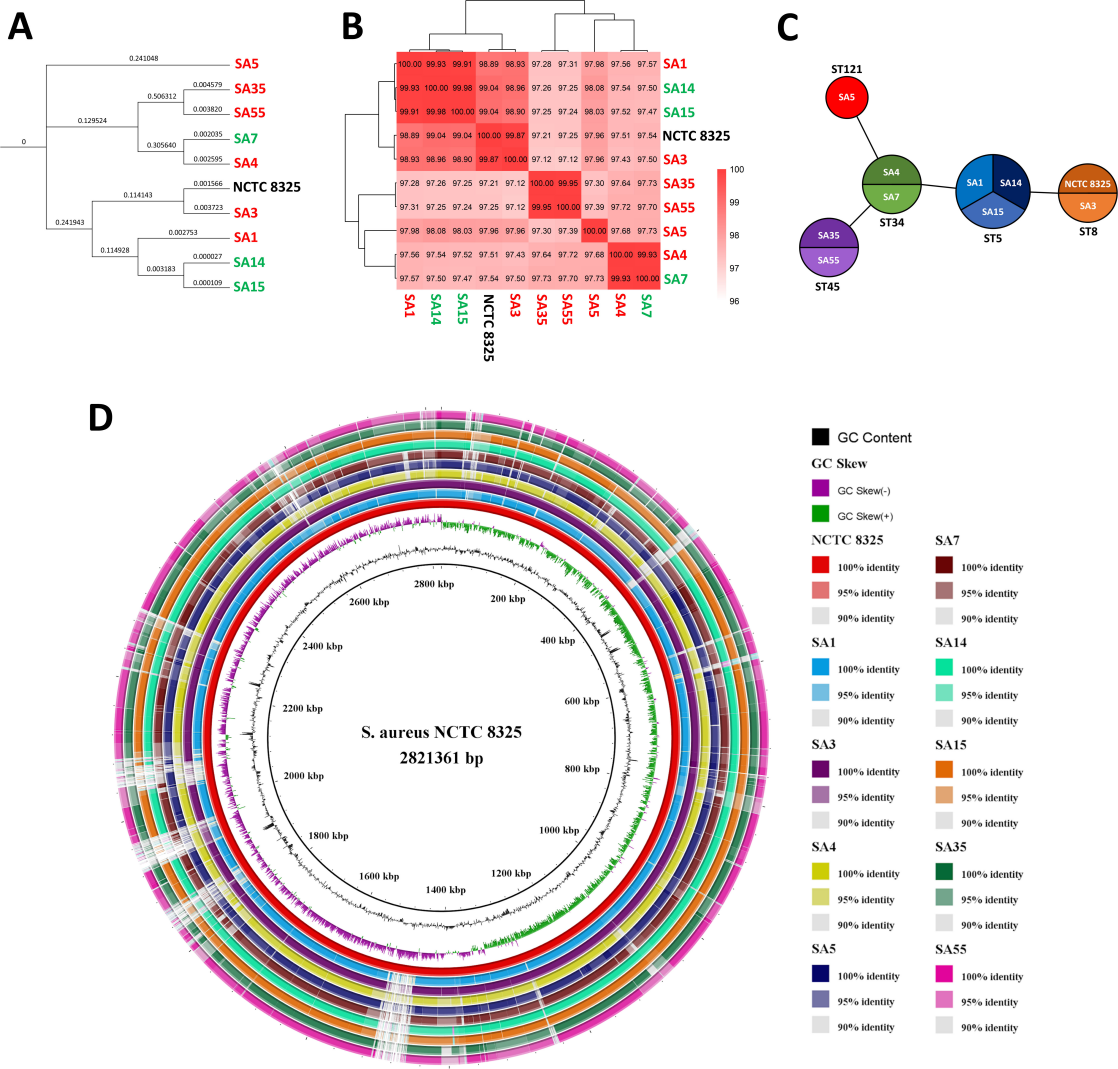
Comparative genomic analysis of 10 *S. aureus* strains analyzed in this study, including core genome SNP-based phylogeny, ANI clustering, MLST typing, and whole-genome alignment. (**A**) Maximum likelihood phylogenetic tree based on core genome SNPs. Branch lengths are proportional to the number of nucleotide substitutions per site, indicating the evolutionary divergence between strains based on core genome SNPs. (**B**) Heatmap of ANI values of the *S. aureus* strains. The clustering of samples based on ANI values is displayed above the heatmap. (**C**) Minimum spanning tree (MST) based on whole-genome MLST profiles of *S. aureus* strains. The MST was generated using the PHYLOViZ online tool (https://online.phyloviz.net/index). Each node in the tree represents a unique sequence type (ST), with node size proportional to the number of genomes sharing that ST. The length between two nodes reflects the genetic distance between the two bounding ST types. (**D**) Sequence alignment of the *S. aureus* strains (SA1, SA3, SA4, SA5, SA7, SA14, SA15, SA35, and SA55) compared against the reference genome *S. aureus* NCTC 8325. The genome map was generated using BRIG. The innermost ring shows the genome size in kilobase pairs (kbp), followed by the GC skew (purple and green), GC content (black), and the reference genome (*S. aureus* NCTC 8325) in red. The outer rings represent the alignment of the individual strains with the reference genome, each depicted in a distinct color. Darker tones in the rings indicate regions of 100% sequence identity, while lighter tones signify reduced sequence identity. Gaps in the alignment highlight regions absent in the corresponding strain’s genome. *S. aureus* strains isolated from clinically healthy women: SA7, SA14, and SA15 (indicated in green); *S. aureus* strains isolated from women with mastitis: SA1, SA3, SA4, SA5, SA35, and SA55 (indicated in red).

A maximum likelihood phylogenetic tree was constructed using core genome SNPs to infer the most likely evolutionary relationships among the strains (Fig. 1A). This phylogenetic tree revealed a division of two main clusters, each containing strains from both mastitis cases and healthy women. Notably, SA5 represented an additional and separate phylogenetic branch. SA14 and SA15, both isolated from healthy women, appeared as the most closely related based on pairwise SNP distance. Interestingly, SA3 clustered closely with *S. aureus* NCTC 8325, a well-known reference strain for *in silico* genomic studies originally isolated from a patient with sepsis.

Species-level identification of the assembled genomes was confirmed through ANI analysis (Fig. 1B). ANI values ranged from 97.12% to 99.98% indicating that all the clinical isolates belonged to the same species. Furthermore, Kraken2 corroborated that the species in all cases was *S. aureus*.

According to their genomic sequence, the analyzed *S. aureus* strains were classified into five multilocus sequence types (ST): ST5 (SA1, SA14, and SA15), ST8 (NCTC 8325 and SA3), ST34 (SA4 and SA7), ST45 (SA35 and SA55), and ST121 (SA5) (Fig. 1C).

### Analysis of core and accessory genes of *S. aureus* strains

**Fig 2:**
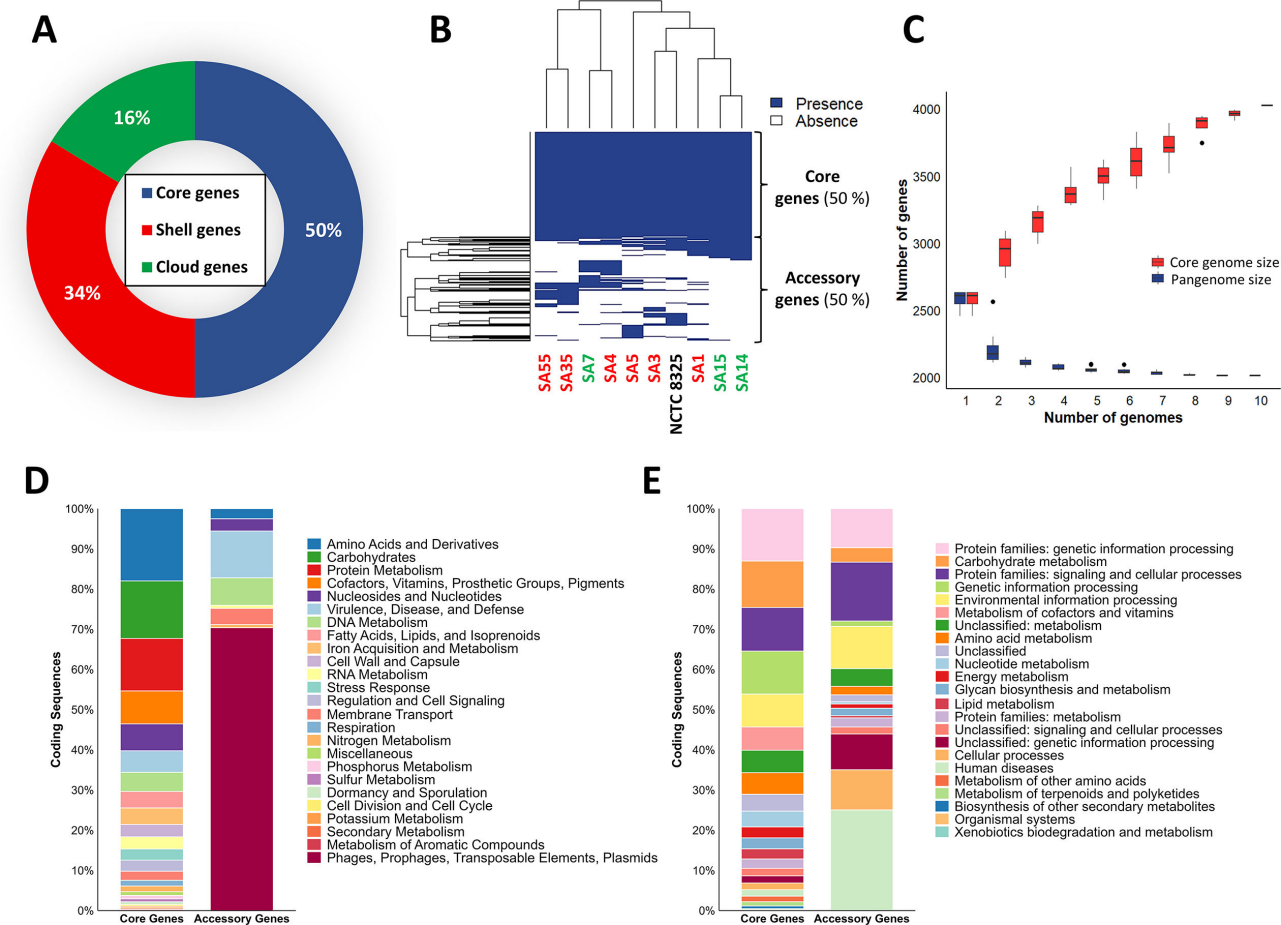
Genome analysis of 10 *S. aureus* strains examined in this study. (**A**) Distribution in genome of core, shell, and cloud genes. (**B**) Heatmap showing the presence and absence of core and accessory genes in *S. aureus* strains. Columns represent the strains, and the rows correspond to all the genes of the strains (4,026 genes). Genes that are present in all strains (shown in blue) belong to the core genome, whereas genes that are present in some strains but absent in others (alternating blue and white cells) are classified as accessory genes. The clustering of samples based on genes’ presence and absence is displayed above the heatmap. (**C**) Development of core genome sizes as the number of sequenced genomes increases. The red boxplots represent the entire set of genes, while core genes are in blue, for each possible combination of n genomes. (**D**) Subsystem categories from RAST for predicted genes within core and accessory gene sets. (**E**) KEGG functional categories of genes identified in the core and accessory gene sets. Each category is represented as a percentage of the total number of genes in the core or accessory gene sets. *S. aureus* strains isolated from clinically healthy women: SA7, SA14, and SA15 (indicated in green); *S. aureus* strains isolated from women with mastitis: SA1, SA3, SA4, SA5, SA35, and SA55 (indicated in red).

A genome map was built to align the genomes of the nine tested strains to the *S. aureus* NCTC 8325 genome (Fig. 1D). Conserved and divergent sections were identified within the analyzed genomes. To improve the comprehension of genetic diversity, adaptability, and virulence of the tested *S. aureus* strains, the distribution and relevance of core and accessory genes in this set of *S. aureus* strains were analyzed for a total of 4,026 CDSs identified. Following the default thresholds established by the Roary pangenome pipeline (38), genes present in at least 95% of the strains were defined as core genes, while those present in fewer strains were considered non-core or accessory genes. The accessory fraction was further subdivided into shell genes (i.e*.*, those present in at least 15% but no more than 95% of the strains) and cloud genes (i.e., those present in no more than 15% of the strains). According to this classification, the core genome comprised 2,013 genes (50% of the total), while the remaining 2,013 genes constituted the accessory genome, including 1,360 shell genes (34%) and 653 cloud genes (16%) (Fig. 2A). A heatmap was generated to visualize the presence/absence of the 4,026 genes across the 10 genomes and the separation between core and accessory genes (Fig. 2B). The number of new genes increased with each additional sequenced genome, while the number of core genes gradually decreased (Fig. 2C). As more genomes were incorporated, core genes experienced a reduction of between 14.1% (with two genomes) and 21.9% (with 10 sequenced genomes), indicating that the proportion of genes shared by all strains decreases as the genetic diversity of the data set increases. On the other hand, the pan genes, which include both core genes and accessory genes, progressively grew with each added genome. The number of new genes increased by between 13.6% (with two genomes) and 56.5% (with 10 genomes), reflecting the discovery of genes exclusive to certain strains that are not present in others, highlighting the genetic diversity among the strains studied.

Functional annotation of genes using RAST revealed distinct subsystems between core and accessory genes (Fig. 2D). Core genes were predominantly associated with essential functions related to the metabolism of amino acids, carbohydrates, lipids, proteins, nucleotides, DNA, RNA, cofactors, vitamins, and iron. Genes implicated in stress response, cellular regulation and signaling, cell wall structure, and membrane transport were also found in this category. In contrast, accessory genes were primarily related to virulence and defense mechanisms. Approximately 70% of the CDSs in this category were ascribed to genes related to the presence of mobile genetic elements such as phages, transposable elements, and plasmids.

The functional annotation of genes with KEGG, and similar to the RAST subsystem analysis, showed that core genes were primarily grouped in categories related to carbohydrate, lipid, protein, and nucleotide metabolism, as well as cofactors and vitamins, processing of genetic information and energy metabolism (Fig. 2E). In contrast, accessory genes predominantly included those associated with human diseases, cellular processes, and genetic information processing. Both RAST and KEGG databases provide complementary insights. RAST gives a broad overview of gene functions by categorizing them into subsystems associated with biological and metabolic processes, while KEGG assigns genes to specific metabolic pathways and molecular interaction networks, offering a more detailed and biochemical understanding of their roles in cellular processes. Together, these databases provide a comprehensive analysis of gene functions, as well as the metabolic pathways and molecular interactions in which they are involved.

### Genetic determinants and antibiotic resistance profiles in *S. aureus* strains

**Fig 3:**
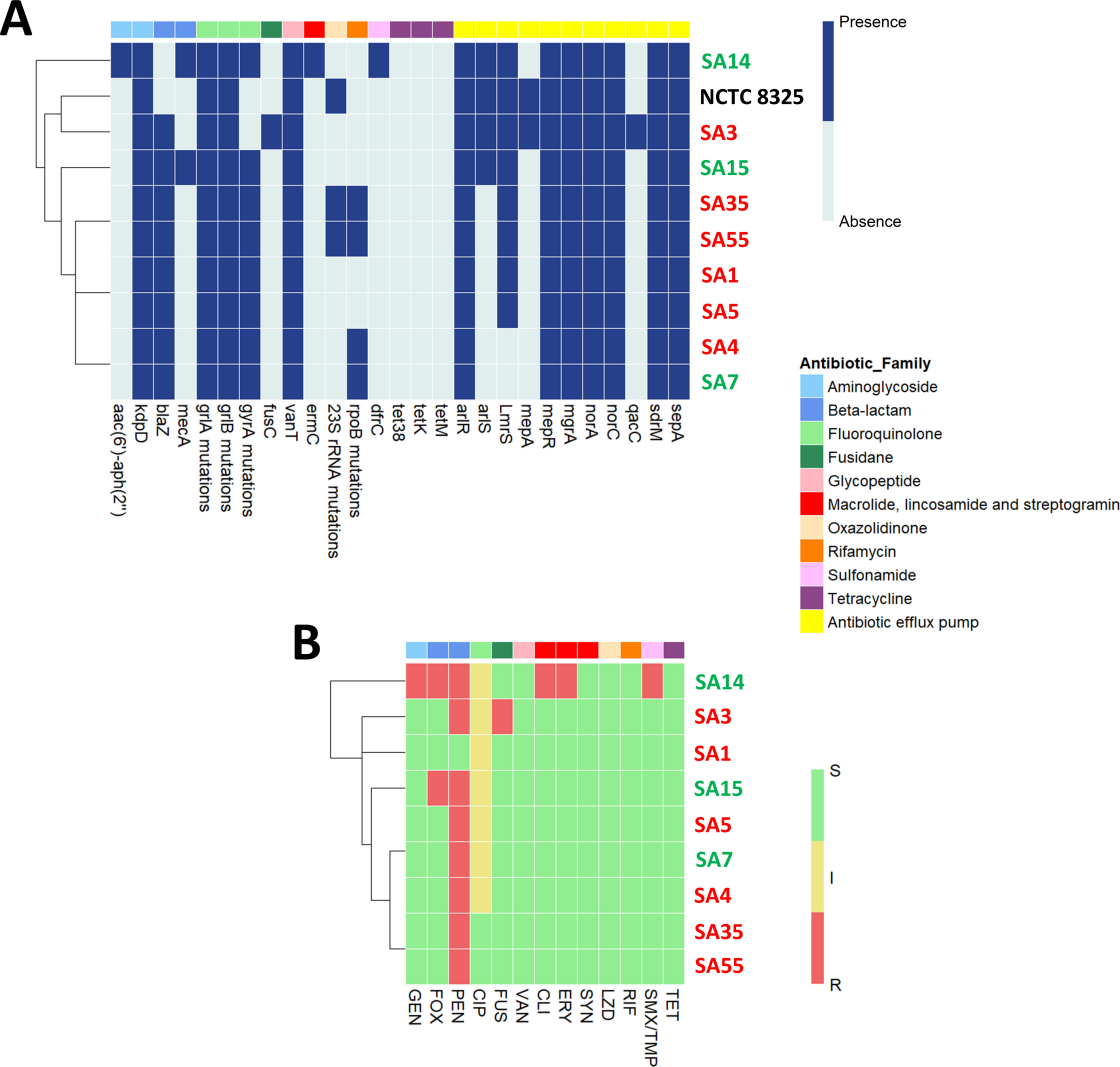
Genetic determinants and antibiotic susceptibility for the 10 *S. aureus* strains analyzed in this study. (**A**) Distribution of antimicrobial resistance genes (ARGs) in the *S. aureus* strains. The heatmap displays the presence (blue) or absence (light blue) of resistance genes. The clustering of strains based on ARGs’ presence and absence is displayed above the heatmap. (**B**) Antibiotic susceptibility of the *S. aureus* strains. The heatmap illustrates resistance profiles categorized as susceptible (S), intermediate (I), and resistant (R) to the antibiotic panel, indicated by green, yellow, and red colors, respectively. The clustering of strains based on antibiotic susceptibility is displayed above the heatmap. Abbreviations used: GEN, gentamicin; FOX, cefoxitin; PEN, penicillin; CIP, ciprofloxacin; FUS, fusidic acid; VAN, vancomycin; CLI, clindamycin; ERY, erythromycin; SYN, quinupristin/dalfopristin; LZD, linezolid; RIF, rifampin; SMX/TMP, sulfamethoxazole/trimethoprim; TET, tetracycline. An additional color legend is used for both heatmaps to denote antibiotic families: aminoglycosides, beta-lactams, fluoroquinolones, fusidanes, glycopeptides, macrolides, lincosamides, streptogramins, oxazolidinones, rifamycins, sulfonamides, tetracyclines, and antibiotic efflux pumps. *S. aureus* strains isolated from clinically healthy women: SA7, SA14, and SA15 (indicated in green); *S. aureus* strains isolated from women with mastitis: SA1, SA3, SA4, SA5, SA35, and SA55 (indicated in red).

The genotypic and phenotypic profiles of ten *S. aureus* strains against different antibiotics were assessed, as antibiotic resistance represents a critical challenge in the control of human mastitis, contributing significantly to the persistence and chronicity of this infection (Fig. 3). Regarding aminoglycosides, the *aac(6′)-aph(2″)* gene, which confers resistance to this antibiotic family, was only present in SA14, supported by its observed phenotypic resistance profile (Fig. 3B). As for the beta-lactam family, all strains except NCTC 8325 and SA14 carried *blaZ*, the β-lactamase coding gene. The phenotypic profile was not entirely consistent with the presence of this gene, as all the strains but SA1 were penicillin resistant. The *mecA* gene, associated with resistance to methicillin, was present in SA14 and SA15 genomes, in accordance with the resistant phenotype to cefoxitin observed in both strains (Fig. 3B). Mutations were identified in *grlA* (100% of strains), *grlB* (100%), and *gyrA* (80%) genes, which might be related to fluoroquinolone resistance. Indeed, all strains exhibited intermediate resistance to ciprofloxacin except for SA35 and SA55, which were sensitive. For the fusidane family, the *fusC* gene was only present in SA3, which was consistent with the observed resistant phenotype in this strain. Although all the strains carried the *vanT* gene, they were susceptible to vancomycin.

In relation to macrolides, lincosamides, and streptogramins (MLSB antibiotics), SA14 was the only strain carrying the *ermC* gene. This correlated with the observed resistance to clindamycin and erythromycin, though this strain was sensitive to quinupristin/dalfopristin. Regarding oxazolidinones, NCTC 8325, SA35, and SA55 presented alterations in the *23S rRNA* gene, which may be related to resistance to linezolid. However, none of the isolates showed phenotypic resistance to this antibiotic. Similarly, although mutations in the *rpoB* gene were found in SA4, SA7, SA35, and SA55, all of them were sensitive to rifampicin. The *dfrC* gene was only present in SA14, which was consistent with the phenotypic resistance to sulfamethoxazole/trimethoprim exhibited by this strain. Finally, none of the strains carried tetracycline resistance genes (*tet38*, *tetK,* or *tetM*), in accordance with the strains’ phenotypic susceptibility to these antibiotics.

Apart from the already mentioned specific genes, the analyzed genomes of *S. aureus* strains carried an important number of antibiotic efflux pump-related genes, including *arlR, mepR, mgrA, norA, norC, sdrM,* and *sepA* (Fig. 3A).

### Virulence factor genes analysis of *S. aureus* strains: genetic determinants and pathogenic potential

**Fig 4:**
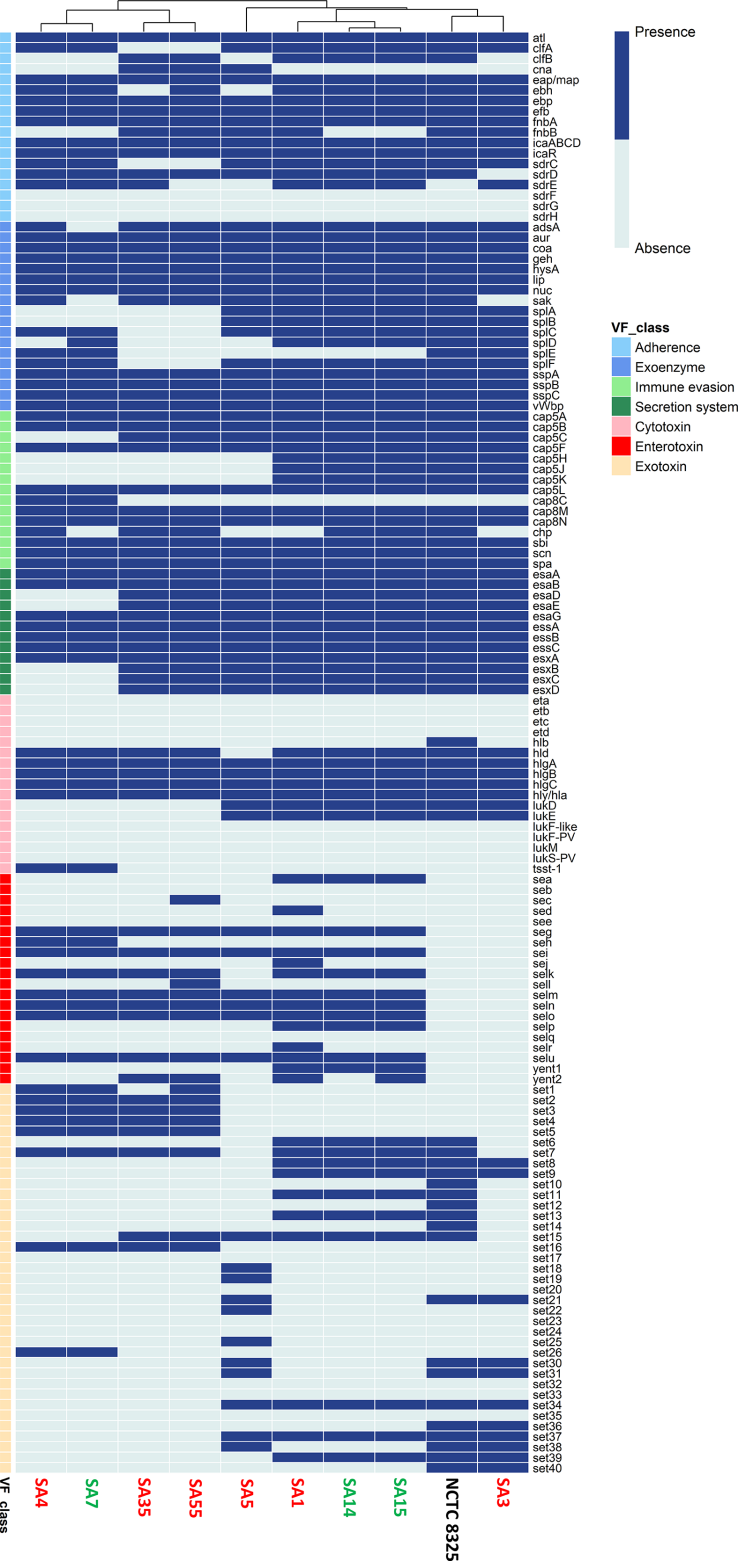
Distribution of different virulence determinants among the 10 *S. aureus* strains analyzed in this study. The heatmap displays the presence (blue) or absence (light blue) of virulence factor genes. The clustering of strains based on the presence or absence of these genes is shown above the heatmap. The classes of virulence factors are indicated with different colors: adherence, exoenzyme, immune evasion, secretion system, cytotoxin, enterotoxin, and exotoxin. *S. aureus* strains isolated from clinically healthy women: SA7, SA14, and SA15 (indicated in green); *S. aureus* strains isolated from women with mastitis: SA1, SA3, SA4, SA5, SA35, and SA55 (indicated in red).

The virulome analysis of the 10 *S. aureus* strains revealed a total of 137 virulence genes covering various functional categories, including adherence, exoenzymes, immune evasion, secretion system, and toxins (Fig. 4). A high presence of key genes related to the ability to adhere to and form biofilms on host tissues, such as *atl, clfA, eap/map, ebh, ebp, efb, fnbA, icaABCD, icaR, sdrC,* and *sdrD*, was observed in most of the studied strains. Interestingly, *cna* and *fnbB* genes, which encode a collagen-binding protein and a fibronectin-binding protein, respectively, were found in most strains isolated from milk from mastitis cases but were absent in those isolated from milk from healthy women (SA7, SA14, and SA15).

Genes encoding extracellular enzymes that contribute to tissue destruction, immune evasion, and degradation of host proteins, enhancing the bacteria’s ability to invade and persist in the host, were found in most of the strains, independently of their origin (Fig. 4). The immune evasion category includes genes that enable *S. aureus* to evade host immune defenses. Notably, *cap5A, cap5B, cap5F, cap5L, cap8M,* and *cap8N,* in addition to *sbi*, *scn,* and *spa*, involved in disrupting immune cell recruitment, complement activation, and antibody responses, were detected in all the strains as occurred with genes essential for the secretion of various *S. aureus* proteins and toxins. Nevertheless, the *tsst-1* gene, associated with toxic shock syndrome toxin, was only present in the genomes of SA4 and SA7, strains which were isolated from milk samples of a woman suffering mastitis and a healthy woman, respectively.

The analysis of enterotoxin genes, related to the impact on the gastrointestinal tract and food poisoning, revealed several key findings. Notably, NCTC8325 (reference strain) and SA3 (isolated from a woman with mastitis) did not contain any enterotoxin genes. Enterotoxin-related genes, such as *sea*, *sec*, *sed*, *seh*, *sej, selk, sell, selp, selr, yent1,* and *yent2*, exhibited variable distribution across the remaining strains, highlighting a rather diverse enterotoxin profile (Fig. 4).

Similarly, the presence of exotoxin genes, some of which behave as superantigens, activating T cells in the immune system of the host in a massive and non-specific manner and resulting in an intensified immunological response, showed wide heterogeneity among the 10 *S. aureus* strains. Notably, no differences were observed between the strains isolated from milk from healthy women and from women suffering from mastitis according to their genetic exotoxin profile. Indeed, SA4 and SA7, isolated from a woman with mastitis and a healthy woman, respectively, exhibited the same exotoxin profile, and the same occurred with SA1, SA14, and SA15.

### Genomic analysis of secondary metabolism in *S. aureus* strains

**Fig 5:**
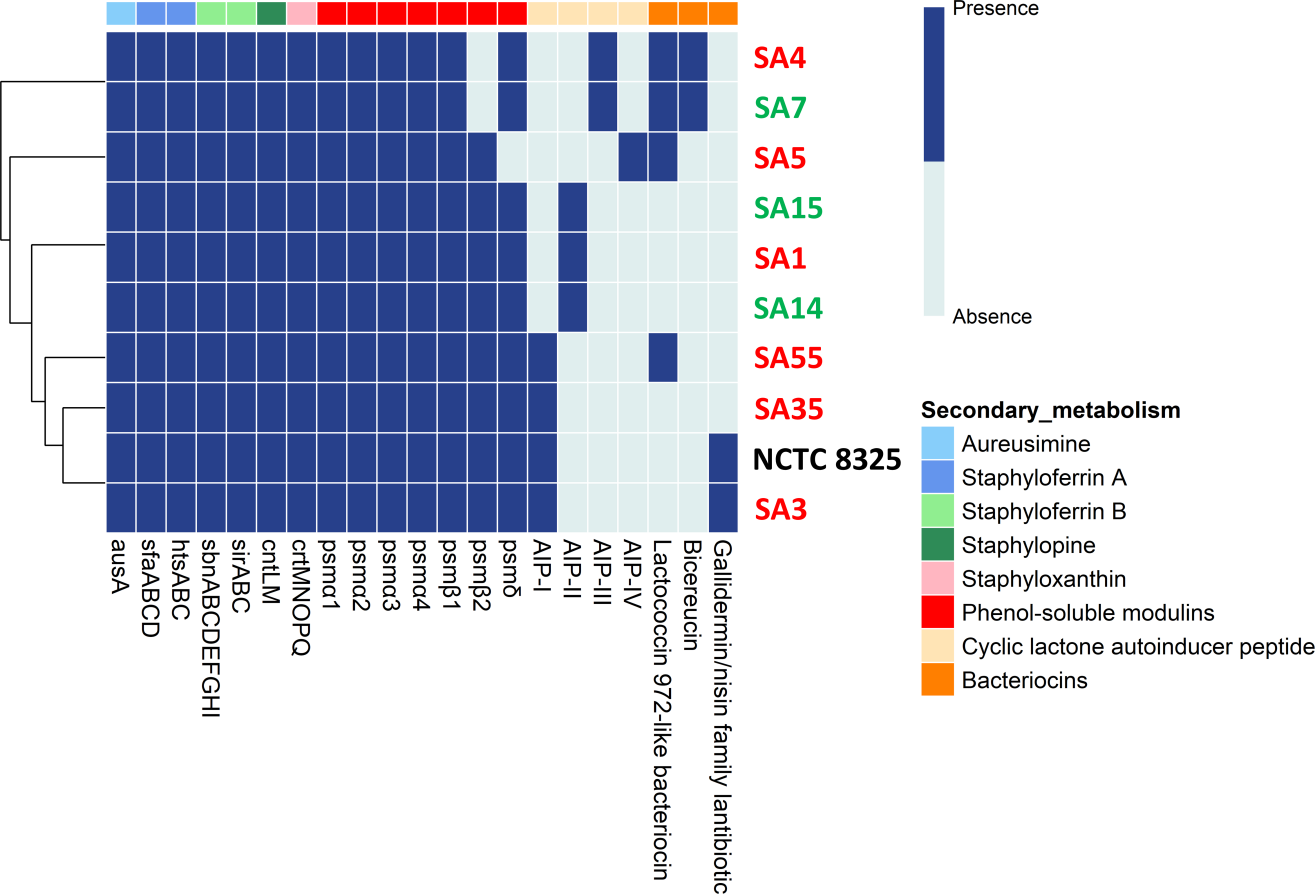
Genetic analysis of secondary metabolism in the 10 *S. aureus* strains analyzed in this study. The heatmap displays the presence (blue) or absence (light blue) of genes or gene clusters involved in the production of secondary metabolites. The clustering of strains based on the presence or absence of these genes or gene clusters is shown at the left of the heatmap. The classes of secondary metabolite genes or gene clusters for the biosynthesis of aureusimine, staphyloferrin A, staphyloferrin B, staphylopine, staphyloxanthin, phenol-soluble modulins, cyclic lactone autoinducer peptide, and bacteriocins are indicated with different colors. *S. aureus* strains isolated from clinically healthy women: SA7, SA14, and SA15 (indicated in green); *S. aureus* strains isolated from women with mastitis: SA1, SA3, SA4, SA5, SA35, and SA55 (indicated in red).

In an attempt to know if the analyzed *S. aureus* strains had specific traits to adapt to different environments and increase their pathogenic potential, the genetic background for various secondary metabolic pathways was examined (Fig. 5). All the tested strains showed a rather similar genetic profile, independently of their origin. All of them had the *ausA* gene, encoding for aureusimines, dipeptides whose functions are not yet fully elucidated, genes involved in the synthesis of the siderophores staphyloferrin A (*sfaABCD*) and staphyloferrin B (*sbnABCDEFGHI*) and their receptors (*htsABC* and *sirABC*, respectively), as well as the genes encoding for the zincophore staphylopine (*cntLM*) and for the staphyloxanthin synthesis (*crtMNOPQ*), a pigment that protects against oxidative stress and UV damage. Genes encoding different subtypes of phenol-soluble modulins (PSMs: PSMα, PSMβ, and PSMδ), peptides that play key roles in virulence through cytolysis, inflammation, and biofilm formation, were also present in all the strains with the exception of SA4, SA7, and SA5, the latter of which lacked several of these genes (Fig. 5).

More differences were found among the *S. aureus* strains regarding the presence of gene clusters involved in the synthesis of four types of autoinducer peptides (AIPs), and the bacteriocins bicereucin, lactococcin 972-like bacteriocin, and a lantibiotic of the gallidermin/nisin family. However, no association was found between the presence of these genetic determinants and the origin of the strain (Fig. 5).

### Mobilome of *S. aureus* strains and its association with virulence and antibiotic resistance genes

**Fig 6:**
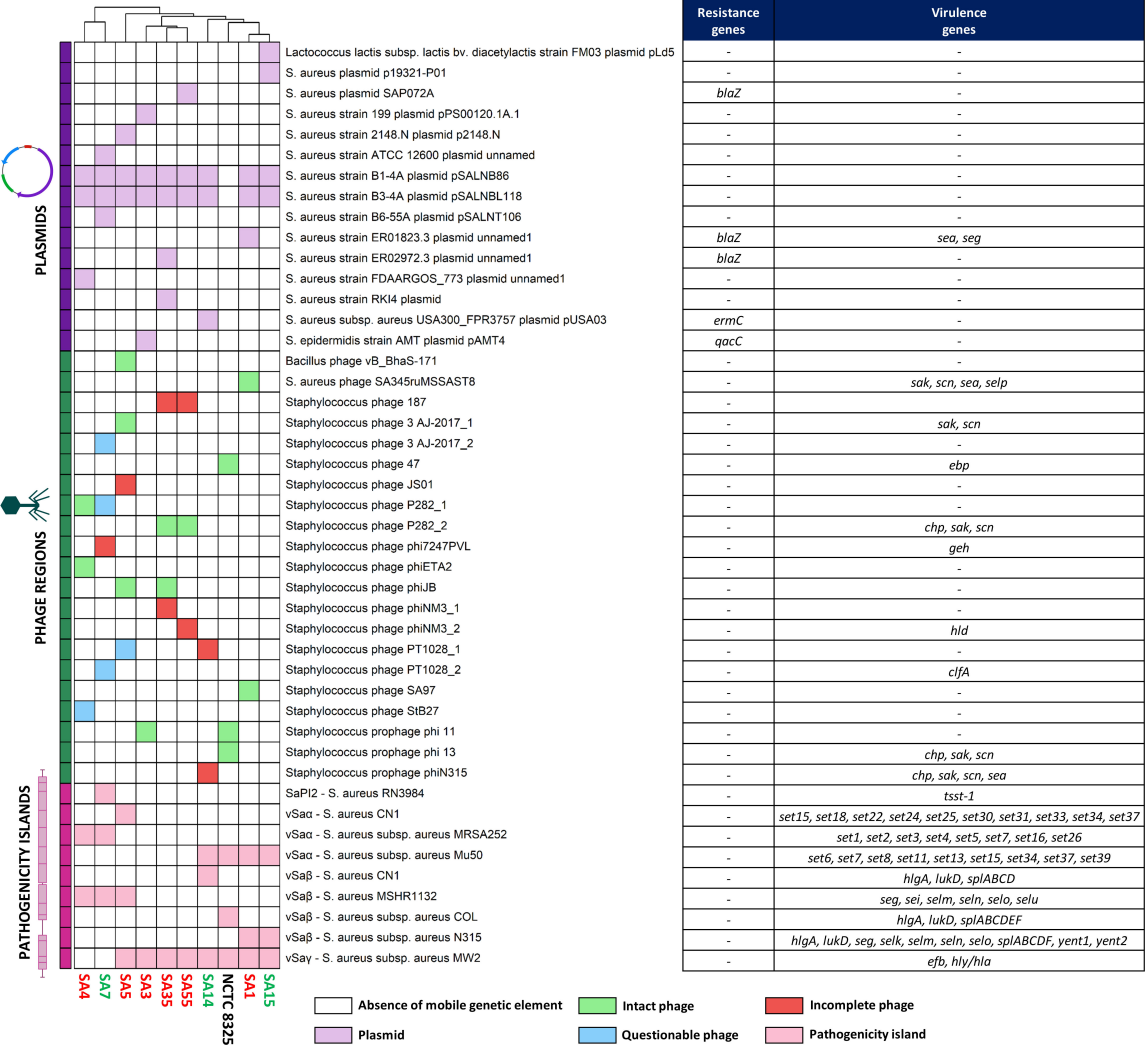
MGEs in the 10 *S. aureus* strains examined in this study are linked to virulence factors and antibiotic resistance genes. The heatmap illustrates the presence (colored cells) or absence (white cells) of mobile genetic elements across the strains. Above the heatmap, strains are clustered based on the presence or absence of these MGEs. Different types of mobile genetic elements are represented by distinct colors: plasmid, intact phage, questionable phage, incomplete phage, and pathogenicity island. To the right of the heatmap, a table lists the antibiotic resistance genes and virulence genes detected within each type of mobile genetic element. *S. aureus* strains isolated from clinically healthy women: SA7, SA14, and SA15 (indicated in green); *S. aureus* strains isolated from women with mastitis: SA1, SA3, SA4, SA5, SA35, and SA55 (indicated in red).

The distribution of mobile genetic elements (MGEs) across *S. aureus* strains and how they correlate with critical genetic features, including antibiotic resistance and virulence, is shown in Fig. 6. Notably, all strains except the reference strain NCTC 8325 harbored at least three plasmids. Some plasmids carry genes conferring resistance to beta-lactam (*blaZ*) and macrolide (*ermC*) antibiotics, as well as virulence factors involved in immune evasion and toxin production (*sea* and *seg*). Importantly, two plasmids*—Staphylococcus aureus* strain B1-4A plasmid pSALNB86 and *Staphylococcus aureus* strain B3-4A plasmid pSALNBL118—were present in all strains isolated from milk. Interestingly, the plasmid pLd5, originally found in *Lactococcus lactis* subsp. *lactis* bv. *diacetylactis* strain FM03, was detected in SA15. This plasmid distribution highlights the genetic diversity and potential clinical relevance of MGEs among the studied *S. aureus* isolates.

Phage-origin sequences were present in all *S. aureus* strains analyzed, comprising 11 intact, 5 questionable, and 7 incomplete phage sequences (Fig. 6). Interestingly, complete phage sequences were absent in the genomes of SA7, SA14, and SA15, isolated from milk of clinically healthy women, whereas all the strains associated with mastitis carried at least one complete phage. None of the detected phages carried antibiotic resistance genes; instead, they were primarily involved in the transfer of virulence genes. All identified phage sequences showed homology to other previously identified phages in *Staphylococcus*, except for vB_BhaS-171, which was present in SA5 and displayed homology to a *Bacillus* phage.

The analysis of the *S. aureus* genomes with the Pathogenicity Island Database allowed us to identify several genomic islands (Fig. 6). Within these regions, antibiotic resistance genes were not detected, being associated exclusively with virulence genes. The SaPI2 pathogenicity island identified in SA7 carried the *tsst-1* gene, encoding toxic shock syndrome toxin-1 (TSST-1), a potent superantigen associated with toxic shock syndrome (58). Additionally, the vSaα, vSaβ, and vSaγ genomic islands were found in various forms across multiple strains, underscoring their widespread presence in *S. aureus* genomes. These regions harbor several staphylococcal enterotoxin (*set*) genes that contribute to toxin production (vSaα), in addition to genes involved in adhesion and hemolysin synthesis (vSaβ and vSaγ, respectively), resulting in cytotoxicity and tissue damage.

### Phenotypic characterization of *S. aureus* strains: early adhesion capability and biofilm-forming ability, and siderophore production

The early adhesion and the biofilm formation capabilities of the *S. aureus* strains were initially studied using polypropylene 96-well plates (Table 2). The initial bacterial population (t_1_) adhering to the inner surface of the wells, after exposing a bacterial suspension (~5 log_10_ CFU/cm²) to the surface at 37°C for 1 h, was similar across all strains, with values ranging from 4.10 to 4.93 log_10_ CFU/cm². After 24 h of incubation (t_24_) at 37°C, representing the biofilm formed, the bacterial counts increased, with SA5 and SA4 showing the highest adherence, with values of 8.29 and 8.06 log_10_ CFU/cm², respectively. Biomass adhered to the surface was separately evaluated by measuring the A_600_ of the material after detachment from the well surface and the A_570_ after crystal violet staining. In both cases, SA5 and SA4 also exhibited the highest biomass adhesion. No significant differences were found between strains isolated from clinically healthy women and women with mastitis for any of these biofilm characteristics.

**TABLE 2:** Early adhesion capability and biofilm formation capacity of *S. aureus* strains^*a*^^,^^*b*^

Strain	Initial adhered population t_1_ (log_10_ CFU/cm^2^)	Viable adhered population t_24_ (log_10_ CFU/cm^2^)	Biofilm biomass (A_600_)	Biofilm biomass (A_570_)
Isolated from clinically healthy women				
SA7	4.85 ± 0.04^a^	7.12 ± 0.24^a^	0.12 ± 0.01^a^	0.39 ± 0.03^ab^
SA14	4.81 ± 0.07^a^	7.46 ± 0.23^ab^	0.13 ± 0.01^ab^	0.38 ± 0.06^ab^
SA15	4.92 ± 0.11^a^	7.53 ± 0.14^ab^	0.14 ± 0.03^ab^	0.38 ± 0.06^ab^
Isolated from women with mastitis				
SA1	4.10 ± 0.04^b^	7.66 ± 0.09^bc^	0.17 ± 0.03^b^	0.54 ± 0.06^b^
SA3	4.69 ± 0.08^a^	7.53 ± 0.08^ab^	0.12 ± 0.00^ab^	0.43 ± 0.10^ab^
SA4	4.71 ± 0.19^a^	8.06 ± 0.10^cd^	0.28 ± 0.03^c^	0.96 ± 0.15^c^
SA5	4.77 ± 0.14^a^	8.29 ± 0.11^d^	0.26 ± 0.05^c^	0.93 ± 0.13^c^
SA35	4.82 ± 0.13^a^	7.54 ± 0.10^b^	0.13 ± 0.01^ab^	0.32 ± 0.08^a^
SA55	4.93 ± 0.14^a^	7.53 ± 0.11^b^	0.13 ± 0.00^ab^	0.36 ± 0.00^ab^

^*a*^ Initial adhered cell population (t_1_) and biofilm characteristics (viable adhered population at t_24_ and attached material measured by A_600_ and A_570_) from *S. aureus* strains in polypropylene 96-well plates.

^*b*^ Different letters above the values indicate statistically significant differences between the means for each parameter studied (one-way ANOVA followed by Tukey-HSD test). For each strain, cell counts were performed in triplicate (*n* = 3), A_600_ was measured in triplicate (*n* = 3), and A_570_ was measured in quadruplicate (*n* = 4).

**Fig 7:**
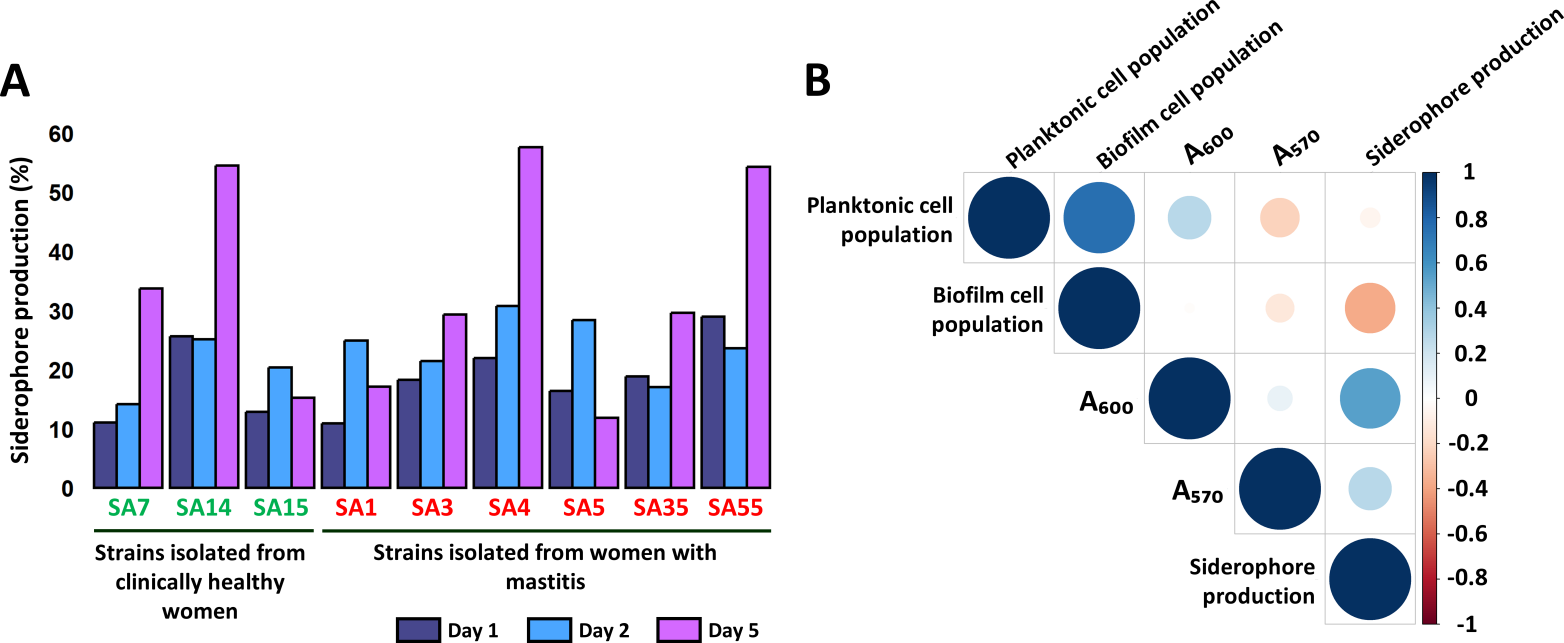
Production of siderophores in the *S. aureus* strains examined in this study. (**A**) Siderophore production (%) at 1, 2, and 5 days of incubation in PMS_7_Ca medium at 37°C, calculated from six biological replicates per strain, with two technical replicates per sample (*n* = 12). (**B**) Correlation analysis between planktonic cell counts, biofilm-forming adhered cells, biomass (A_600_), crystal violet-stained adhered material (A_570_), and siderophore production (%) for the *S. aureus* strains. *S. aureus* strains isolated from clinically healthy women: SA7, SA14, and SA15 (indicated in green); *S. aureus* strains isolated from women with mastitis: SA1, SA3, SA4, SA5, SA35, and SA55 (indicated in red).

Varying levels of siderophore production were observed under iron-limiting conditions for all *S. aureus* strains, depending on the strain and the incubation time (Fig. 7A). At day 1, SA14 and SA55 exhibited the highest siderophore production (25.63% and 28.95%, respectively). As the incubation progressed to day 2, an increment in the siderophore production was registered for most of the strains, with SA4 and SA5 (associated-mastitis strains) producing 30.79% and 28.38%, respectively. By day 5, a considerable increase in siderophore production was observed in certain strains, particularly SA4, SA14, and SA55, which reached 57.60%, 54.49%, and 54.27%, respectively. To further explore potential relationships between the different measured variables, a correlation analysis was performed (Fig. 7B). This analysis revealed that the planktonic cell population showed a significant positive correlation with biofilm cell population (*r* = 0.75, *P* < 0.05). Additionally, siderophore production was positively correlated with biomass (A_600_), which represents the combined planktonic and biofilm cell populations (*r* = 0.54, *P* < 0.05), suggesting that increased siderophore production could correspond to higher biomass levels.

## DISCUSSION

*S. aureus* is the main causative agent of acute lactational mastitis, but, at a much lower extent, it may also be detected in milk samples from healthy women, using both culture-dependent and culture-independent methods (16, 17, 59–64). The dual role of *S. aureus*, as a colonizer of some body sites (e.g., skin, throat, and intestine) in asymptomatic people and as an opportunistic pathogen responsible for a wide spectrum of infections, has long been recognized. Nonetheless, the bacterial factors underlying its capacity to shift between these roles and its specific interactions with the host remain poorly understood (13). In this study, we compared six strains of *S. aureus* isolated from the milk of women with mastitis (SA1, SA3, SA4, SA5, SA35, and SA55) and three strains from the milk of clinically healthy women (SA7, SA14, and SA15), with the aim of identifying differences in genetic and phenotypic characteristics that could explain such duality.

The analysis of *S. aureus* genomes revealed that approximately 50% of the genes are “core genes” shared by most strains, coding for essential functions such as carbohydrate, lipid, and protein metabolism, DNA replication, and cellular regulation. The remaining genes constitute the “accessory genome,” which is associated with virulence, host adaptation, and environmental versatility. In addition to the high proportion of accessory genes, significant variability within this fraction was observed, as reported in other studies (65–68). The genetic plasticity of the accessory genome likely provides adaptive advantages, enabling *S. aureus* to thrive in diverse ecological and clinical niches, enhancing its ability to cause infections.

The maximum likelihood phylogenetic analysis based on core genome SNPs revealed no clear clustering of strains from women with mastitis compared to those from healthy women. Similarly, ANI comparisons and MLST typing also showed a high degree of similarity among the strains, supporting the lack of distinct grouping based on clinical origin, which confirms results from previous studies (14, 66). This observation can be attributed to the tropism of these strains for the mammary gland, as all were isolated from human milk, suggesting a predisposition to colonize this location regardless of the presence of an active infection. The ability of these strains to colonize the mammary gland without causing symptoms or inducing mastitis suggests a complex adaptive mechanism that enables *S. aureus* to balance commensalism and pathogenesis depending on host and microbial factors.

The genome analysis of these *S. aureus* strains revealed a high number of virulence determinants covering various functional categories, including adherence, exoenzymes, immune evasion, secretion systems, and toxins. Virulome analysis has proven fundamental for understanding the functional differences between *S. aureus* strains. A set of genes involved in host tissue adherence and biofilm formation, including *atl, clfA, eap/map, icaABCD, icaR, sdrC,* and *sdrD*, was present in all strains (69, 70). Notably, specific genes such as *fnbB* and *cna* were exclusively identified in strains from women with mastitis, suggesting their role in conferring a competitive advantage for colonizing mammary tissue and enhancing *S. aureus* pathogenicity, fostering epithelial cell invasion, as shown previously (71, 72). Exoenzyme-related genes such as *adsA*, *aur, coa, geh, hysA, lip, nuc, sak, sspA, sspB,* and *sspC,* and immune evasion-related genes such as *cap5A, cap5B, cap5F, cap5L, cap8M, cap8N, sbi, scn,* and *spa* were also found in most strains, indicating that *S. aureus* possesses efficient machinery to degrade host tissues and mechanisms to circumvent the host immune response (73–75). Most *S. aureus* strains also displayed genes related to secretion systems, like *esaA, esaB, esaG, essA, essB,* and *essC*, and genes implicated in hemolysin and leukotoxin production (*hld, hlgA, hlgB, hlgC,* and *hly/hla*), implying a shared ability to secrete virulence-related proteins and potential to cause cellular damage (73, 76). Overall, the presence of shared genes across many strains highlights their key role in the adaptation and survival of *S. aureus* in different host environments, beyond their relationship with mastitis. Nonetheless, their presence may enable *S. aureus* to act as an opportunistic pathogen in favorable situations.

On the contrary, enterotoxin and exotoxin-related genes, which could impact infection type and progression, showed greater variability, although no clear distinction could be made between strains from healthy women and those with mastitis, as previously observed in relation to *S. aureus* strains causing bovine mastitis (76, 77). Some exotoxins behave as superantigens, activating T cells in the host in a massive and non-specific manner, resulting in an intensified immunological response (78). The observed genetic differences underscore the need for further studies to explore the functional impact of individual MSCRAMMs and toxins and their relationship with virulence in mastitis.

The analysis of secondary metabolism in *S. aureus* provides critical insights into how this pathogen adapts to hostile environments, such as the human body, and secures survival and virulence advantages. In our study, all strains carried genes linked to the production of aureusimines, staphyloferrins, staphylopine, and staphyloxanthin. These siderophores facilitate metal acquisition under conditions of limited availability, playing important roles in regulating virulence and host-pathogen interactions (79–84). Phenotypic analyses revealed that all *S. aureus* strains secrete siderophores at varying levels when cultured individually in defined media. *In vivo*, siderophore production may change substantially when growing in mixed bacterial communities in iron-limited environments such as milk, reflecting dynamic microbial interactions and adaptation to resource scarcity (85). Additionally, genes responsible for the synthesis of PSMs were identified in most strains. PSMs promote biofilm formation and trigger inflammatory responses, and variations in their production could influence the persistence of chronic infections (86).

In *S. aureus*, the sophisticated Agr quorum-sensing system, which regulates the expression of various virulence factors based on bacterial density, is of particular relevance (87). This system is controlled by AIPs that, upon reaching a threshold concentration, activate signaling cascades that induce RNAIII transcription. RNAIII is the primary regulator of virulence factors such as hemolysins, proteases, and toxins. The Agr system also facilitates biofilm dispersion, enabling *S. aureus* to transition from an adherent to a more invasive and virulent one. The main types of AIPs (AIP-I, AIP-II, AIP-III, AIP-IV) not only activate their own Agr system but also inhibit the systems of competing strains, fostering inter-strain competition (88). However, our study did not identify a clear relationship between the presence of AIP genes and mastitis. In contrast, previous findings have associated the Agr III group with increased biofilm formation and certain diseases like toxic shock syndrome (89, 90).

Interestingly, genes encoding lactococcin 972-like bacteriocin, bicereucin, and a gallidermin/nisin family lantibiotic, not previously reported in *S. aureus*, were detected in this study (91–95). The secretion of these antimicrobial peptides may confer a competitive advantage by eliminating competing strains and establishing a niche favorable for the bacteriocin-producing strain. For *S. aureus*, this ability could contribute to the development of mastitis by promoting dominance in the mammary gland. Nevertheless, conventional antimicrobial activity assays conducted in this study did not reveal the production of any antimicrobial compound by these *S. aureus* strains, suggesting that either the genes are not functional or that the laboratory conditions used did not allow their expression.

The *S. aureus* mobilome is key to understanding the evolution of its virulence and antibiotic resistance. Our results indicate that mobile genetic elements, including plasmids, phages, and pathogenicity islands, could promote horizontal gene transfer, enabling rapid adaptation of the strains (96). Notably, antibiotic resistance genes such as *blaZ* and *ermC* were exclusively identified in plasmids, highlighting the crucial role of these elements in resistance to beta-lactams and macrolides, confirming recent studies (97, 98). Virulence genes related to adhesion, exoenzyme production, and toxin synthesis were distributed across plasmids, phages, and pathogenicity islands, reflecting the genetic complexity and adaptability of *S. aureus* (99, 100).

An important finding was the presence of complete phages in the genomes of all mastitis-associated strains, in contrast to incomplete or questionable phages found in strains isolated from clinically healthy women. Complete phages may confer a competitive advantage to mastitis-associated strains, particularly under stress conditions like an active infection, by entering the lytic cycle and eliminating competing bacteria within the same niche (101, 102). This is consistent with studies in bovine mastitis, where *S. aureus* strains isolated from milk from mastitis cases were used to isolate a wide variety of bacteriophages, which were detected in all tested dairy samples, highlighting the high prevalence of phages in mastitis-related environments (103). Although direct evidence in human milk is still limited, a metagenomic analysis has reported the presence of viral sequences in samples from women with mastitis, suggesting a potential role of phages in the microbial dynamics of the disease (15). Additionally, the identification of a phage of *Bacillus* origin and plasmids derived from *S. epidermidis* highlights the potential for horizontal gene transfer between different species coexisting in shared environments, such as milk or skin (97). These horizontal gene transfer events, especially prevalent in mastitis-associated strains, reinforce the ability of *S. aureus* to acquire and disseminate resistance and virulence genes, thereby enhancing its pathogenic potential. This emphasizes the relevance of such mechanisms in the evolutionary and adaptive success of *S. aureus* in both clinical and community settings. Interestingly, none of the detected phages carried antibiotic resistance genes; rather, they were primarily involved in the transfer of virulence genes. This illustrates the role of phages in increasing the pathogenic potential of specific *S. aureus* strains.

The absence of significant differences regarding the presence of genetic determinants between *S. aureus* strains isolated from women with mastitis and those from healthy women, as observed in this study, may be attributed to the multifactorial nature of infection development, which is not solely dependent on bacterial traits. Both groups of strains share the same environment—breast milk—and the progression to disease may be influenced by host-related factors, such as host immune responses. For instance, genetic polymorphisms influence the activation of Toll-like receptors in antigen-presenting cells. Moreover, an imbalance in the production of pro-inflammatory cytokines (e.g., IL-1α/β, IL-6, IL-8) and anti-inflammatory cytokines (e.g., IL-4, IL-10, IL-13) may predispose certain mothers to develop infections (104, 105). Other external factors, such as the use of antibiotics during pregnancy and lactation or the frequent use of breast pumps, may alter the mammary microbiota and compromise its defensive capacity (106). Consequently, addressing the prevention and treatment of mastitis requires a comprehensive approach that considers not only the biology of the pathogen but also host-specific factors and the impact of medical interventions.

The treatment of clinical mastitis traditionally relies on antibiotics, but the increasing antimicrobial resistance in *S. aureus* complicates therapeutic success. In this study, the genotypic profile of resistance for aminoglycosides, fusidic acid, sulfonamides, and methicillin and sensitivity to tetracyclines [presence of *aac(6′)-aph(2″*), *fusc*, *dfrC*, and *mecA* genes and absence of *tet38*, *tetK,* or *tetM*] corresponded to the phenotypic resistance profiles. Interestingly, the *mecA* gene, which confers methicillin resistance, a key antibiotic used to treat *S. aureus* infections, and resistance to cefoxitin, an indicator for detecting methicillin resistance (107), was only found in two strains (SA14 and SA15). In contrast, some discrepancies were found regarding the fluoroquinolone family, beta-lactams, and the MLSB family, as it has been observed in previous studies (98, 101, 107). The observed concordances and discrepancies between phenotypic and genotypic profiles confirm the complexity of antimicrobial resistance mechanisms in *S. aureus* and emphasize the need for detailed analysis to inform therapeutic strategies. In addition, the obtained results indicate that antimicrobial resistance predictions based on *in silico* genome analysis should always be accompanied by antibiotic sensitivity testing to draw reliable conclusions (101).

Antimicrobial efflux pumps were found in all *S. aureus* strains, but these elements must be positively regulated to contribute to resistance (108). Other studies have also reported high prevalence of these elements in *S. aureus* strains, including fluoroquinolone resistance genes (*norA* and *norC*) and their regulators (*mgrA* for *norC* and *arlS* for *norA*), a major facilitator superfamily efflux pump (*LmrS*), a multidrug efflux pump (*sdrM*), a repressor for a multidrug export protein (*mepA*) and its regulator (*mepR*), and the disinfectant resistance gene (*sepA*), which has also been shown to induce biofilm formation (98, 101, 109).

In addition to the antimicrobial resistance of *S. aureus*, another significant challenge in treating mastitis is the formation of biofilms within the galactophore ducts during infection. These organized multicellular structures protect bacteria from antibiotics and the host immune system, contributing to the chronicity of the infection (110–112). This highlights the urgent need for innovative strategies to combat persistent and recurrent infections caused by *S. aureus* strains in mastitis patients.

One of the limitations of this study was the relatively low number of *S. aureus* strains included in the genomic comparison, which restricts the statistical power and limits the ability to draw broader conclusions about the genetic and functional differences underlying mastitis. Work is in progress to expand the data set by including a larger number of well-characterized *S. aureus* strains from the milk of mastitis and healthy women, which will allow for more robust and statistically meaningful comparative genomic analyses.

### Conclusions

The analysis of key genomic features, including the presence of virulence factors, resistance genes, and biofilm-forming capabilities, revealed or confirmed some mechanisms by which *S. aureus* contributes to the pathogenesis of mastitis. Genotypic and phenotypic differences between strains isolated from the milk of women with mastitis and those from healthy women were primarily associated with the presence of *fnbB* and *cna* genes, which encode fibronectin and collagen adhesins, as well as complete bacteriophage genomes in the mastitis-related strains.

## Data Availability

The authors declare that the data supporting the findings of this work are available within the paper. Genomes are available at NCBI under BioProject ID PRJNA1200689. Should any raw data files be needed, they are available from the corresponding author upon request.
